# Best Practices and Lessons Learned for Action Research in eHealth Design and Implementation: Literature Review

**DOI:** 10.2196/31795

**Published:** 2022-01-28

**Authors:** Kira Oberschmidt, Christiane Grünloh, Femke Nijboer, Lex van Velsen

**Affiliations:** 1 eHealth Cluster Roessingh Research and Development Enschede Netherlands; 2 Biomedical Signals and Systems Group University of Twente Enschede Netherlands

**Keywords:** action research, eHealth, best practices, lessons learned

## Abstract

**Background:**

Action research (AR) is an established research framework to introduce change in a community following a cyclical approach and involving stakeholders as coresearchers in the process. In recent years, it has also been used for eHealth development. However, little is known about the best practices and lessons learned from using AR for eHealth development.

**Objective:**

This literature review aims to provide more knowledge on the best practices and lessons learned from eHealth AR studies. Additionally, an overview of the context in which AR eHealth studies take place is given.

**Methods:**

A semisystematic review of 44 papers reporting on 40 different AR projects was conducted to identify the best practices and lessons learned in the research studies while accounting for the particular contextual setting and used AR approach.

**Results:**

Recommendations include paying attention to the training of stakeholders’ academic skills, as well as the various roles and tasks of action researchers. The studies also highlight the need for constant reflection and accessible dissemination suiting the target group.

**Conclusions:**

This literature review identified room for improvements regarding communicating and specifying the particular AR definition and applied approach.

## Introduction

The way health care is organized and executed is of great societal concern, as it affects our quality of life. Hence, health care systems and eHealth technologies used to support health care should be designed in a way that meets the needs and expectations of their stakeholders. One way of doing this is through action research (AR). According to Bradbury and Lifvergren [1], AR in health care “seeks to (1) improve patient experiences and the health of populations, (2) reduce the per capita cost, (3) improve the work life of those who deliver care, and (4) bring health care providers into circumstances that allow for continuous learning together with patients.” AR has been used as a research framework in nursing and health care, for example, to improve the quality of patient care and investigate changes in action [2]. AR is a collaborative approach, where people affected by the change envisioned in AR become active members of the research team. AR is often used in the design of eHealth systems. However, existing literature reviews of AR in eHealth predominantly focus on the development of new frameworks [3-5] but not on how eHealth AR is currently carried out. Therefore, this literature review outlines the state of the art of AR in eHealth design.

eHealth projects cover a wide variety of topics and technologies and can therefore greatly benefit patients, professionals, and many other health care stakeholders. However, to gain the most from eHealth systems and technologies, it is crucial that they match with what is needed in practice [6]. To ensure such a match, Van Gemert-Pijnen and colleagues [6] suggest, among other things, working together with relevant stakeholders in all stages of the project, implementing the study results in practice, and continuously evaluating the process. Similarly, co-design has been mentioned as a useful technique for creating eHealth systems that suit the needs of the end users [3]. These ideas fit well with the principles of AR, which will be outlined below.

**Figure 1:**
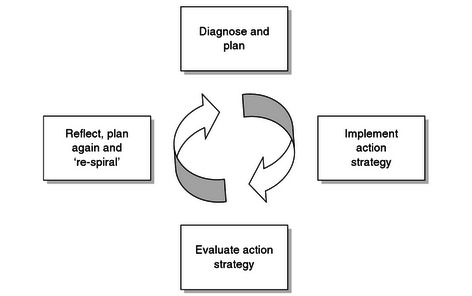
Action research cycles (adapted from Kurt Lewin [7] by Williamson and colleagues [2]).

Definitions of AR have changed over the years. AR originated with Kurt Lewin [7], who described it as several consecutive circles of planning, action, and reflection. These cycles are shown in Figure 1, developed by Williamson and colleagues [2]. In later definitions, the cyclical nature of AR remains one of its key features. Reason and Bradbury [8], who build on Lewin’s work, define AR as research that (1) involves stakeholders not only as participants but also as members of the research team, (2) consists of (at least) 1 cycle of planning, action, and reflection, (3) establishes direct changes, and (4) then evaluates those changes in and with the community. Their work [8] includes many interesting examples of AR from various fields. Furthermore, Bradbury and colleagues defined 7 “choice points for quality in action research” [9], criteria that can be used to plan, conduct, report, and assess AR projects*.*

Within AR, different variations exist, such as action design research (ADR) or participatory action research (PAR). Usually, there is agreement on the main principles of AR explained earlier, but some authors or groups emphasize some aspects over others. For example, as the name suggests, ADR incorporates elements of design research into AR [10], whereas PAR highlights the involvement of the community [11]. For a more detailed overview of the similarities and differences between some of these approaches, see Williamson et al [2] or Coghlan and Brannick [11].

In general, AR and AR approaches such as ADR are similar to participatory design (PD) approaches that are used in human computer interaction (HCI) research. However, AR emphasizes reflection on and learning from the process that was carried out, whereas the main aim of PD is to create a solution [12]. AR, as opposed to PD, does not start with a clear goal of what needs to be developed but defines this throughout the process together with stakeholders. Additionally, AR is more immersive and calls for stakeholder involvement for a longer period of time due to its iterative cycles [13]. Nevertheless, in some cases, studies that are described as PD-related ones also meet Reason and Bradbury’s criteria [8] for AR [14]. Hayes [12] argues that AR and HCI research can supplement each other, as both often provide solutions on a local scale. As Hughes [15] describes, there is no standard way of implementing AR in health care due to the broadness of the field. Instead, there is a variety regarding the why, how, and with whom AR in health care is carried out [15,16]. For example, levels of stakeholder engagement and the context in which AR takes place can vary [16]. Other differences among AR studies include the topic, country, project duration, main target group, and methods used. Therefore, these aspects are considered in this review. The purpose of this review is to give an overview of the current literature on eHealth AR and summarize the best practices and points of improvement for future eHealth AR projects. Special attention is paid to the contextual variables of the research (eg, setting, duration, number of stakeholders), as this is expected to influence the outcomes, best practices, and points of improvement of a study. To provide an overview of AR in eHealth, this literature review addresses the following subquestions:

1. What is the context of AR eHealth projects?
2. How do eHealth AR studies define and operationalize AR?
3. What are the best practices for conducting AR in concrete eHealth studies?
4. What are the lessons learned from conducting AR in concrete eHealth studies?

## Methods

### Study Selection and Screening

**Figure 2:**
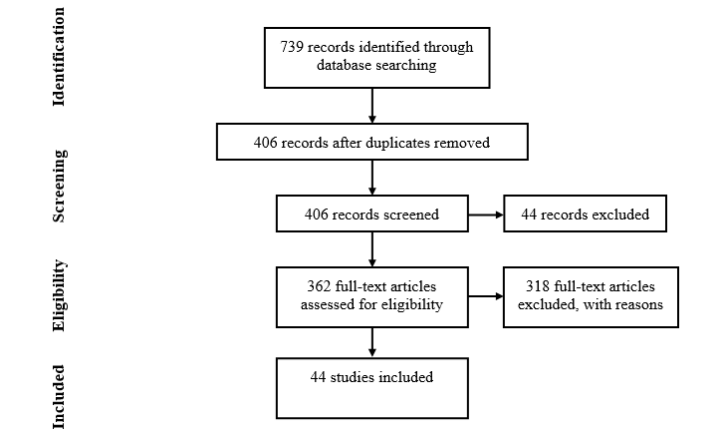
Inclusion flowchart of the literature search and screening process.

The search was carried out in June 2020. PubMed, Scopus, and Google Scholar were searched using combinations of the search terms “action research” or “participatory design” and “eHealth,” “health technology,” “digital health,” or “telemedicine.” PubMed was chosen for its extensive medical database, and Scopus and Google Scholar were chosen as large scientific databases. Searching for “action research” turns up articles that include similar and related keywords like “participatory action research,” “action design research,” or “action-based research.” “Participatory design” was included as a search term because PD has significant overlap with AR, and both are sometimes used to supplement each other. The list of synonyms for “eHealth,” although not exhaustive, is expected to cover the various facets of the field. The initial search yielded 739 results. Articles were included if they (1) used and explicitly mentioned AR and (2) were about eHealth or health technology. Papers were excluded if they (1) were not written in English, (2) only included a study protocol but did not report results, or (3) only included a review of other articles. Full-text screening of the same 15 articles was performed by 2 authors (KO and CG); the authors discussed whether to include the studies until an agreement was reached. Next, the first author screened the full texts of the remaining articles, with some exceptions where a second opinion was necessary. These were again discussed between the first and second authors until an agreement was reached. Ultimately, 44 articles were included, reporting on 40 different projects. Figure 2 shows the flowchart of the inclusion process.

### Data Extraction

For each study, the definition of AR that was provided by the authors, and the related AR approaches that they cited (if any) were extracted. Additionally, information about contextual variables of the study was derived. Specifically, we identified the topic, country, organizational context, project duration, types of stakeholders involved, the main target group of the research, and methods used. The types of involved stakeholders were grouped according to the framework described by Schiller et al [17], in which they define the main stakeholder categories as the public, policy makers, and governments, the research community, practitioners and professionals, health and social service providers, civil society organizations, and private businesses. Finally, the best practices and lessons learned were derived. The best practices and lessons learned were activities that could move forward and benefit the AR project, without necessarily being recognized as standard components of AR. The difference between what was seen as a best practice and as a lesson learned was based on the timing and reporting of these actions. An activity was labeled as a best practice if researchers already planned their project with this in mind (eg, mentioning it in the description of the methods). On the other hand, lessons learned were those points that researchers came to know during their project, which were reported mainly in the discussion section. From the first 5 articles, the best practices and lessons learned were extracted by 2 authors (KO and CG), and they compared their results. The remaining data were extracted by 1 author (KO) in consultation with the second author where a second opinion was necessary. Furthermore, 5 authors published not 1 but 2 papers about their project. For these papers, the same study context was described whereas the definition of and approach to AR and the best practices and lessons learned were reported separately, as these sometimes differed between the articles. A reflection on 2 projects was included in 1 article. In this case, each project context was reported separately whereas only 1 AR definition and approach as well as one set of best practices and lessons learned were outlined.

### Synthesis

A general overview of all the included studies describing the AR approach, AR definition, and contextual variables was obtained. The contextual variables (topic, location, target group, stakeholders, duration, and methods used) were categorized. Furthermore, the studies were mapped in a matrix based on the study duration and the types and number of different stakeholders that participated in the study. The contextual data were coded and categorized inductively. To identify which AR approach was the most used, the citation frequency of each approach in the included studies was recorded. Furthermore, the cited AR approaches that were available were accessed and checked for cross-referencing. All cited AR definitions were mapped to show the relationship between them. The AR definitions used, best practices, and lessons learned were coded by 1 author (KO). The best practices and lessons learned were coded individually first and then combined for both categories.

## Results

### Context

The setting of the included studies was described based on 6 categories (topic, location, duration, involved stakeholders, target group, and methods). Multimedia Appendix 1 presents all the categories and the description of the setting for each study. The most common aspects of each category will be discussed below.

#### Topic

We identified 9 broader categories of the research topics in the 44 included studies (see Table 2.1 in Multimedia Appendix 2 for the full list). The most common were home care and telemonitoring, and health promotion and education (both n=8), followed by electronic medical records and health information systems (n=7), and mental health services (n=5).

#### Location

The studies were set in 21 different countries, Australia being the most common (n=5) followed by the United States (n=4) and Canada, Sweden, and the United Kingdom (all n=3). Some studies from nonwestern countries, like Tanzania or Colombia were included, but no country was represented more than once or twice. Within the different countries, studies took place in various contexts, the most prevalent of which were rural areas (n=6) and hospitals (n=5). All contexts and countries can be found in Tables 2.2 and 2.3 of Multimedia Appendix 2.

#### Target Groups

Among the 44 studies, 2 studies explicitly focused on 2 different target groups at the same time, whereas all other studies had 1 main target group. In most cases, the target groups were patients (n=11). Of these, the most common group was patients with cancer (n=3). There were 6 studies each focusing on clinicians as well as children and young adults, and 5 studies targeted older adults (see Table 2.4 in Multimedia Appendix 2 for the full list of target groups).

#### Stakeholders

In many cases, several stakeholders were included in the study, up to 6 different types of stakeholders included in some cases. In summary, 20 different types of stakeholders were involved (see Table 2.5 in Multimedia Appendix 2 for the full list). Health care workers (n=18) and patients and their representatives (n=12) were involved the most, followed by governmental bodies (n=9) and general nonmedical staff members (n=8). When clustering these stakeholder types according to the framework defined by Schiller and colleagues [17], the largest group consisted of practitioners and professionals (n=48), followed by members of the public (n=38). Policy makers and government bodies (n=13), the research community (n=10), private businesses (n=6), and civil society organizations (n=3) were represented less often. The only group that was not represented at all included health and social service providers.

#### Duration

**Figure 3:**
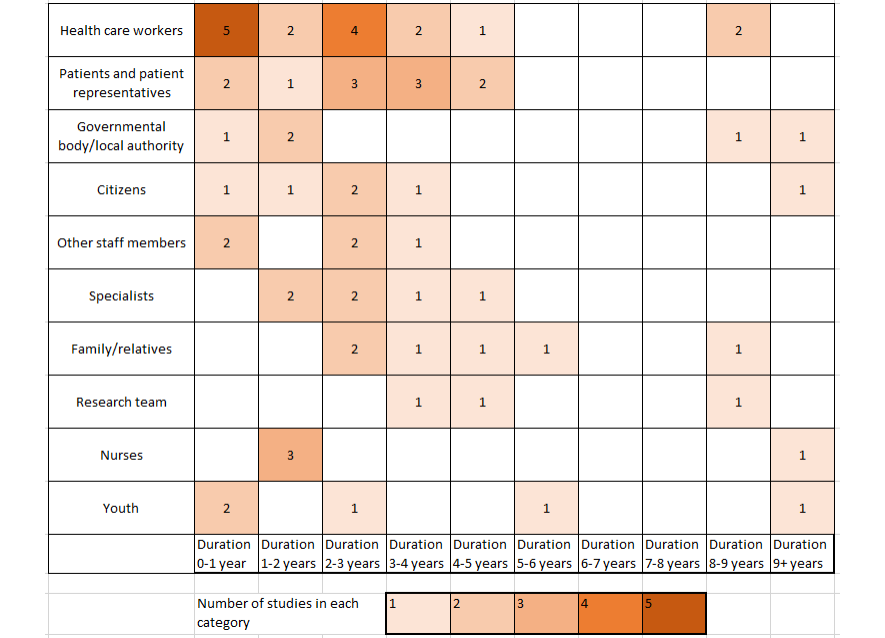
Heat map showing the most commonly involved types of stakeholders against the project duration.

Not all of the 44 studies reported the duration of the project (n=7). Studies that did report the duration (n=33) lasted from a few months (n=5) to more than 10 years (n=2). The majority (n=13) of these studies reported a project duration between 2 and 3 years, and the average project duration was 2.7 years. Figure 3 shows the distribution of the 10 most frequently involved types of stakeholders for the different project durations in the 33 projects that reported the project duration. Stakeholder types are shown in the order of how many times they were involved in total; however, because some studies did not report project durations, the numbers in this graph differ from those described above. The 2 biggest stakeholder groups, health care workers and patients, were rarely, or in the case of patients even not at all, involved in long-term studies.

**Figure 4:**
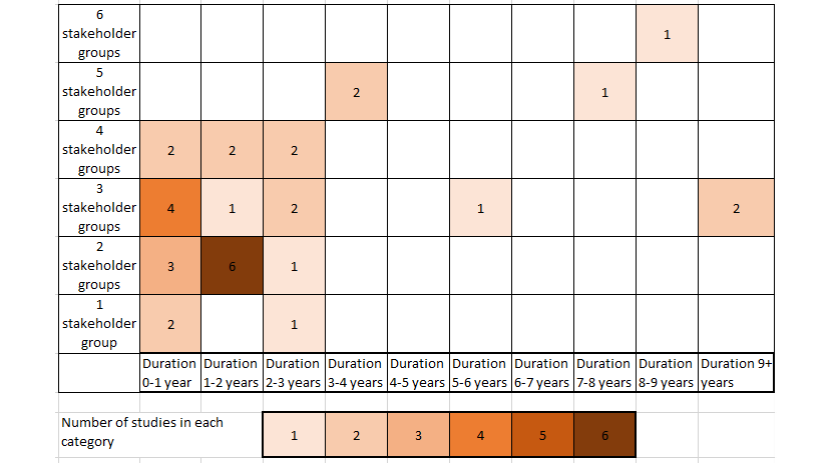
Heat map showing the number of stakeholders involved against the project duration.

In Figure 4, the study duration is mapped against the number of different stakeholders that were involved in each of the 33 projects that reported a project duration. Studies that did not report the overall project duration are not included in the figure. Most of the included studies lasted for up to 2 years, including 2 or 3 stakeholder groups. There are some longer studies including more stakeholder groups.

#### Research Methods Used

As mentioned earlier, AR is a framework that does not advise the use of a single methodology, and studies can therefore include a variety of different research methods. Most of the 44 included studies indeed used several methods, with some studies employing up to 6 different methods*.* Interviews were used most frequently (n=24), followed by focus groups (n=22), workshops (n=14), and surveys (n=13). On average, studies used nearly 3 different methods (average 2.8). All methods can be found in Table 2.6 of Multimedia Appendix 2.

### AR Definitions

The articles contained 44 definitions of AR. They could be grouped according to 4 different aspects that they emphasized. First, 21 studies emphasized that in AR projects, practitioners and other stakeholders become (co)researchers (n=21). Second, AR is a cyclical process that includes different stages (n=19). Third, 14 studies described how AR focuses on solving a practical issue and aims to extend research knowledge. The fourth aspect was that AR takes place in a community setting (n=10). Further, 2 studies included 3 of these aspects in their definitions, and only 2 other studies mentioned all 4 aspects. Most studies included either 1 (n=16) or 2 (n=17) of the aspects, whereas 7 studies included none of these points in their definition or did not at define AR in detail. Table 1 provides an overview of the number of mentions per aspect and the studies mentioning these aspects.

**Table 1:** Number of mentions and studies mentioning the aspects of the AR definition.

Aspect of the AR definition	Number of articles that define AR including this aspect, n (N=44)	References
Practitioners and other stakeholders being (co)researchers	21	[18-38]
Cyclical process including different stages	19	[23,24,26,27,30,31,34,35,38-48]
Aiming to solve a practical problem and extend academic knowledge	14	[20,21,25,30,32,36-39,42,43,49-51]
Research taking place in a community setting	10	[19,20,23,28-30,38,52-54]

### AR Approaches

**Figure 5:**
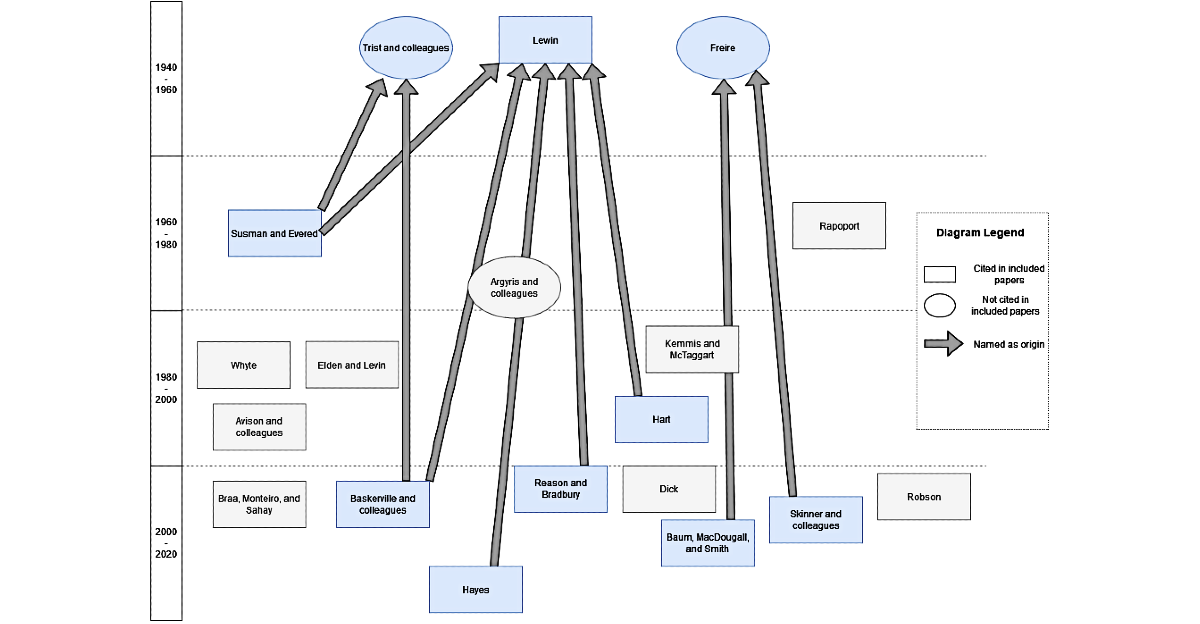
Overview of the action research approaches referred to in the included articles, indicating those papers that are mentioned as “the origin” of action research. Studies that either name an approach as being the origin of action research, or are being named as such, are highlighted in blue for better readability.

**Figure 6:**
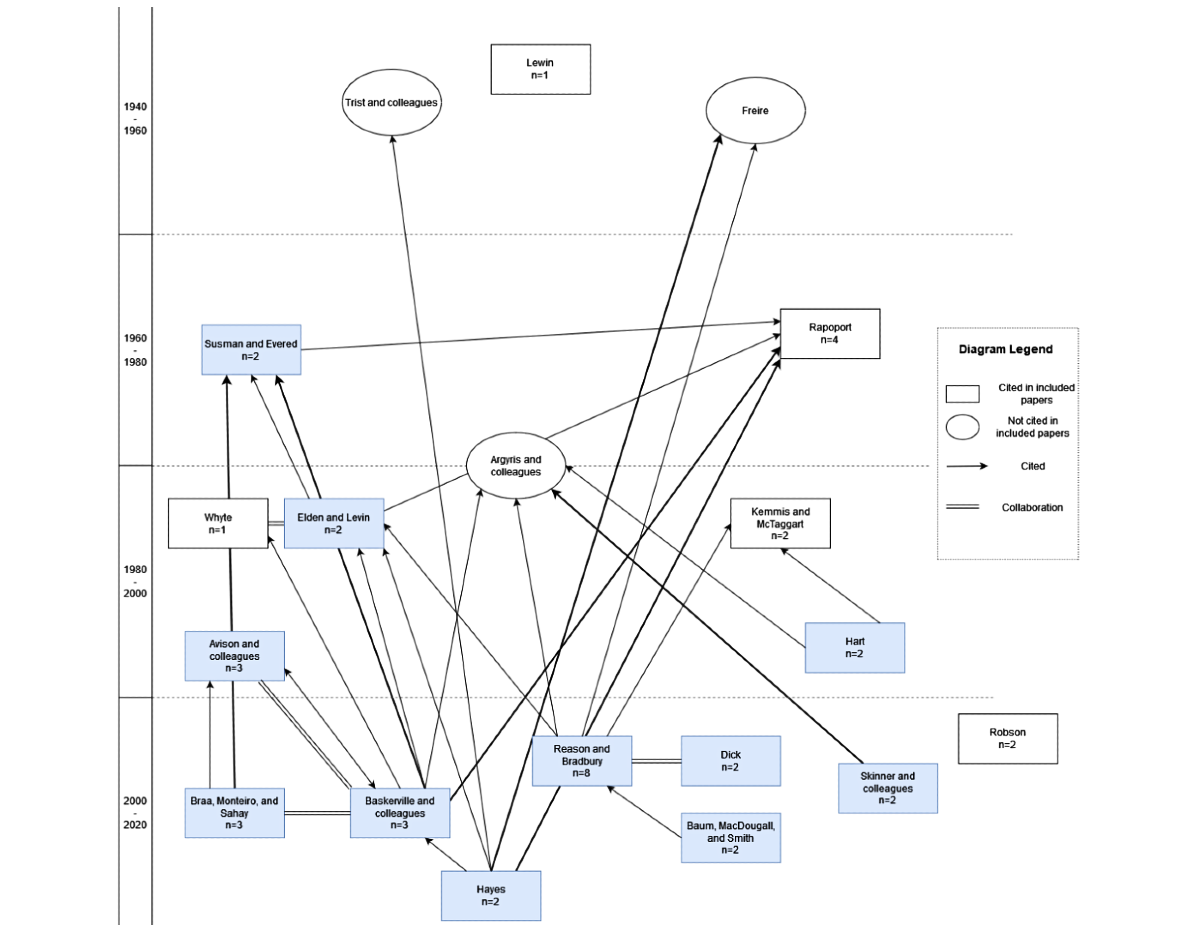
Overview of action research approaches referred to in the included articles. Arrows indicate citations between the action research approach papers. The number of times that the articles included in this review cited each approach is indicated in the box. We have used different arrow thicknesses for better readability. Blue boxes indicate those papers that were available and checked for citations.

Table 2 gives an overview of the AR approaches that were cited at least twice in the included articles. The AR approach was not cited in 4 studies. In some cases, different papers from the same authors were cited; however, as these eventually described the same approach, the citation count was added up. The most commonly cited approach was that proposed by Reason and Bradbury [8]. As described earlier, the key elements of this approach are that AR (1) involves stakeholders as coresearchers, (2) consists of plan, act, and reflect cycles, (3) makes a change in practice, and (4) evaluates the said changes in and with the community. Overall, most definitions share these main aspects but differ in terms of the aspects that are particularly emphasized. For example, Baskerville and colleagues [55] highlight the duality of practical work and scientific knowledge, whereas Baum and colleagues [56] underline the need for reflective practice that includes all stakeholders. Figures 5 and 6 depict the cited approaches in more detail. There are 3 independent researchers or groups that are mentioned as being the origin of AR, namely Lewin [7], Trist and colleagues [57], and Freire [58]. Wherever the origin of AR was mentioned, some cases have named 2 of these, as observed in Figure 5. The cited AR approaches also frequently refer to each other and sometimes authors collaborate with each other, for example on books about AR (see Figure 6). There are no very distinct groups conducting their own AR, but the different AR groups are often connected and build upon each other’s work.

**Table 2:** Overview of the most cited action research approaches in the included articles per author or research group, including the number of citations.

Author(s)	Number of author citations	References	Action research approach paper(s) describing these approaches
Peter Reason and Hilary Bradbury	8	[23,28,35,37,42,44]	[8,59,60]
Robert N. Rapoport	4	[35-37,39]	[61]
David Avison and colleagues	3	[37,39,49]	[62]
Richard L. Baskerville and colleagues	3	[29,38,43]	[55,63,64]
Jørn Braa, Eric Monteiro, and Sundeep Sahay	3	[18,53,54]	[61]
Stephen Kemmis and Robin McTaggart	3	[19,44,51]	[66,67]
Fran Baum, Colin MacDougall, and Danielle Smith	2	[24,52]	[56]
Bob Dick and colleagues	2	[35,44]	[68,69]
Max Elden and Morten Levin	2	[32,52]	[70]
Colin Robson	2	[20,38]	[71]
Harvey A. Skinner, Oonagh Maley, and Cameron D. Norman	2	[45,72]	[72,73]
Gerald I. Susman and Roger D. Evered	2	[38,43]	[74]
Elizabeth Hart	2	[33]	[75,76]
Gillian R. Hayes	2	[47,77]	[12]

### Best Practices and Lessons Learned

As previously described, an activity was identified as a “best practice” if researchers already planned their project with this in mind (eg, mentioning it in the description of methods). Lessons learned were those points that researchers came to know during their project. These were mostly reported in the discussion section. In total, 85 best practices and 66 lessons learned were identified, which were clustered into 22 categories of best practices and 16 categories of lessons learned. Among the 44 papers, 3 papers did not indicate any best practices that they followed, whereas 12 papers did not include any identifiable lessons learned. There were 8 overlapping categories, identified as best practices in some articles and as lessons learned in others. These will be discussed in more detail below.

#### Best Practices

The identified best practices in the 44 studies were most often related to the use of a specific method (n=9), namely personas (n=2), world café, journey mapping, role play, scenarios, case studies, design cards, and mixing different types of data collection methods (all n=1). Other best practices were a continuous evaluation of the project and a reflection on the process by the research team (n=8). The importance of establishing active contact between researchers and stakeholders and raising the confidence and skills of stakeholders was emphasized by 7 studies. The improvement of stakeholder skills mainly referred to research and analytical skills, allowing stakeholders to set up their own studies or continue the work after the project was finished. There were several specific suggestions to improve the regular project team meetings, for example, to always use the same agenda or to share a common area (office space) to make contact easier. Some other best practices concern the reporting and presentation of outcomes (n=6). The complete list of best practice categories can be found in Table 3.

**Table 3:** Overview of all best practice categories and number of mentions per category (N=44).

Best practices category				Number of mentions, n
**Process**				
	**Recommends specific method**			
		Personas	2	
		World Café	1	
		Journey mapping	1	
		Role play	1	
		Scenarios	1	
		Case study	1	
		Design cards	1	
		Abstract vs personal methods of data collection	1	
	Continuous evaluation and reflection		8	
	**Report or present results**			
		Share resources and findings (on the internet) allowing others to benefit from it	4	
		Present findings to the community or target group in a suitable manner	2	
	Start with close examination of context (observation and literature)		5	
	Agile development and Scrum		3	
	**Combining action research with randomized controlled trials (RCTs)**		2	
		Combining these 2 approaches	1	
		Keeping the line between stakeholders and researchers blurred and not performing RCTs	1	
	Gradual scaling up		2	
	Immediately resolve problems and apply lessons learned		2	
**Stakeholders and relationships**				
	Frequent or regular (face-to-face) meetings, active contact (eg, shared space), and same transparent agenda		7	
	Raising stakeholder confidence and skills (eg, analytical skills so that they can set up their own studies)		7	
	Clearly defining the role of each partner (equal involvement is not always good)		5	
	Finding committed stakeholders with intrinsic motivation (to carry on with the project after the researchers have left)		5	
	Reference group (with technical, juridical, and clinical expertise)		4	
	Stepping into each other's shoes (experiencing the other’s tasks and familiarizing oneself with what the other does)		3	
	Investing in relationship between partners (also nonwork activities)		3	
	Adapting methods or schedules to the needs of stakeholders		3	
	Neutral position of the researcher (no steering or predetermined outcomes, serving as a communication link instead)		3	
	Patient- and stakeholder-generated content (eg, personas)		2	
	Different disciplines		2	
**Context and environment**				
	Living labs as context for action research		2	
	Actively encouraging pilot participation		2	
	Paying attention to economic or business values		3	

#### Lessons Learned

Apart from the best practices, the lessons learned from each study were identified. The most common lessons learned were increasing stakeholder knowledge and skills (n=8) and continuous evaluation of the project and reflection on the process (n=6). Both of these had been identified as best practices in other articles (more on this overlap below). Recommendations for the use of specific methods were also common (n=5). Lessons learned regarding reporting, adapting the project to fit the needs of stakeholders, fostering a welcoming environment, and the questionable replicability of the research were each mentioned 4 times. All lessons learned are shown in Table 4.

**Table 4:** Overview of all lessons learned categories and number of mentions per category (N=44).

Lesson learned category				Number of mentions, n
**Process**				
	Continuous reframing or renegotiation (flexibility), baby steps		6	
	**Recommend specific method**			
		Field work	1	
		Randomized controlled trial	1	
		Case study	1	
		Action circles	1	
		Fun methods (quiz, game, puzzle) as learning opportunities	1	
	**Reporting**			
		Open source	2	
		Higher level of sophistication necessary	1	
		Also include nonproject target group	1	
		Integration of literature	3	
		Regular meetings to check on progress and motivate the stakeholders (reality check)	2	
		Triangulation of data to decrease biases	2	
	**End of an action research project**			
		Accompanying stakeholders until they find that the process is done	1	
		Action research leading to other collaborative activities	1	
	Commitment to action research necessary (eg, through specific funding)		1	
	Ethical restrictions		1	
	Immediate reflection impossible		1	
**Stakeholders and relationships**				
	Raising stakeholder confidence and skills, knowledge sharing		8	
	**Tailoring to the needs of stakeholders**			
		Including action research in work schedule	1	
		Researchers taking over some of the stakeholders’ usual tasks to make schedule less busy	1	
		Adequate feedback methods	1	
		Identifying unique strengths	1	
	Investing in relationship between partners		3	
	Accepting that participation is different for everyone and can change over time		3	
	**Communication**			
		Language barrier	1	
		Finding a common language	1	
	Enthusiastic local ”champion” to start the project and help keep people motivated		2	
	Involving authorities or local government (address issues at multiple levels)		2	
	Actively breaking down power structure		1	
**Context and environment**				
	Fostering a positive, welcoming environment for change		4	
	Questionable replicability		4	
	Active researcher involvement and presence in environment		2	
	Drawing attention to external influences		1	
	Ethical issues		1	
	Diffusion of innovation		1	
	Organizational expectations		1	

#### Overlapping Best Practices and Lessons Learned

As stated earlier, some aspects were identified as best practices in some articles and as lessons learned in others. In total, we identified 7 such overlapping aspects. Overall, the most mentioned aspect was the importance of raising stakeholder skills and confidence (n=15, where best practices= 7 and lessons learned=8). Many articles reported the need for stakeholders to learn new skills, for example related to academic research, or the need to be convinced about their ability to perform these tasks. Almost all the studies that reported this as a best practice or lesson learned involved health care professionals as stakeholders. Other commonly mentioned points were recommendations for specific methods, even though the suggested methods differed (n=14, where best practices=9 and lessons learned=5) and there was continuous reframing and evaluation of the project (n=14, where best practices=8 and lessons learned=6). Continuous reframing often referred to the iterations of planning, action, and evaluation in AR projects. Studies that described this mostly did not include this cyclical nature of AR in their definition of it. In total, there were 10 recommendations regarding the reporting and presentation of results (best practices=6 and lessons learned=4), for example calling for open and accessible publishing of outcomes. The best practices and lessons learned included recommendations about meeting regularly (n=9, where best practices=7 and lessons learned=2), adapting to the needs of stakeholders (n=8, where best practices=3 and lessons learned=5), and investing in the relationship between partners (n=6, where best practices=3 and lessons learned=3).

#### Chronology of Overlapping Best Practices and Lessons Learned

When observing the publication timeline, most of the overlapping aspects appeared as a lesson learned in earlier publications, and then as a best practice in papers published at a later point in time. This was the case regarding stakeholder skills, appearing as a lesson learned in 1999 [33] and as a best practice in 2016 [25]; continuous reframing of the project was a lesson learned in 2003 [19] and best practice in 2009 [42]; further, having regular meetings was a lesson learned in 2006 [72] and a best practice in 2018 [27], and adapting the research to stakeholder needs was a lesson learned in 2007 [32] and a best practice in 2016 [77]. Such a clear timeline could not be seen for accessible reporting, appearing as a lesson learned in 2017 [78] and a best practice in 2007 [45], and the relationship between partners appearing as a lesson learned in 2017 [36] and as a best practice in 2008 [38].

## Discussion

### Principal Results

To identify recommendations on how to conduct AR in eHealth studies, this literature review analyzed the setting, AR description, and best practices and lessons learned in 44 studies. The most important recommendations from this review, which will be discussed in more detail below, are as follows: actively raising stakeholder skills and confidence; fulfilling multiple roles and tasks as a researcher; fostering constant reflection and evaluation; ensuring open and accessible dissemination; reporting in a more structured and comprehensive way.

These recommendations are not exclusively related to eHealth, despite them being derived from a review of eHealth AR studies. Hence, it is possible that the recommendations are also relevant for AR in various other fields. Therefore, where possible, examples from different disciplines are discussed below to explain or supplement a recommendation.

#### Stakeholder Skills and Confidence

Being involved in a project as coresearcher can potentially increase stakeholders’ confidence, besides teaching them new skills [79]. However, this does not happen automatically. Similar to our findings, the narrative review conducted by Harrison and colleagues [80] also identified educating the research team as the most important task when stakeholders are involved in health care research. Nevertheless, there is limited research on how skill training for stakeholders could look like, and this can vary greatly between studies. Stakeholders in some eHealth studies might need to learn content-related information [81], whereas other studies require methodological or statistical skills [54]. Researchers should provide adequate training and material for their project and encourage stakeholders to make use of it. The studies included in this review that recommended stakeholder skill training almost exclusively worked with health care professionals. The relationship between recommending skill training and working mainly with health care professionals remains unclear. A possible explanation could be that other stakeholder groups in other studies already had the necessary skills and thus did not require any additional training. Another possibility is that other stakeholders were not given the same roles that health care professionals held, and therefore, they did not need skill training. Finally, as we will discuss later, reporting of AR activities was not always very extensive. Thus, stakeholders outside the health care sector were possibly trained, and these studies did not report on this aspect. Generally, not all participants prefer the same level of engagement in a project, and researchers should respect these preferences [82].

#### Tasks and Roles of the Researcher

Different aspects of the role and tasks of the researcher in an AR project are discussed. Brydon-Miller and Aragón describe the many different tasks that action researchers need to fulfil as their “500 hats” [83]. These are not specific to eHealth studies, but they can occur in any AR study. As researchers and stakeholders have many varied duties, their roles are not fixed and might change over the course of the project [19]. One main task of the researchers that continues throughout the project is the need to foster a welcoming environment for all stakeholders [42]. Researchers should also be present and actively involve themselves at a higher level than that needed in non-AR projects [38]. Additional AR-specific tasks for the researchers include investing in partner relationships [35] or breaking down power structures [28]. Generally, AR studies demand more self-reflection and awareness from the researchers than other projects and researchers should keep this in mind when entering an AR project.

#### Constant Reflection

The importance of continuous reframing and evaluation of the project was emphasized in several studies. Although evaluation is 1 of the AR cycles, studies providing recommendations on this topic rarely included this in their definition of AR. Owing to the lack of reports on AR cycles, which will be discussed below, it is unclear if these studies still followed the AR cycles without reporting on them. However, sometimes, it seems that periodic planned evaluation is not enough. Instead, the participants need to regularly reflect on the current status of the project and their role in it. Therefore, new AR projects should create suitable spaces for evaluation and reflection in ways that fit the projects and stakeholders. This is especially important because reflection can become difficult once a person is in the middle of the project [49]. Holeman and Kane [53] emphasize that reflection should not only take place within the project, but it should also be explicitly reported to help other researchers. If action researchers take reflection seriously and include honest evaluations in their publishing, the AR community members can learn from each other. Additionally, researchers and other stakeholders within the project learn and benefit from constant reflection [9].

#### Accessible Dissemination

Another important aspect concerns paying attention to open and understandable dissemination of results within the community and among researchers. Action researchers need to communicate findings to the academic world while also finding ways to inform the target group about the project in ways that suit the target users’ needs. An example of open and accessible dissemination can be found in Canto-Farachala and Larrea [83]. They present the results of their AR project regarding territorial development on an interactive website, allowing others to learn from their work. However, it seems that accessible reporting is still not the norm in AR, as Avison and colleagues [62] describe that many AR studies are generally “published in books rather than as articles. Action researchers have large and complicated stories to tell.” Future AR projects should attempt to narrate their stories in such a way that others can learn from them.

#### Comprehensive Reporting

The different way of describing AR studies also leads to another issue, incomplete and elusive reporting. Although most studies did provide at least a short description of what they saw as AR, 7 studies provided no definition at all. Additionally, there were only 4 studies that included 3 or all of the 4 aspects of the AR definition in their description. Even the most mentioned aspects appeared in less than half of the included papers. Even though most papers did cite an AR approach of definition, some did not. In combination with the often-limited descriptions of AR, this makes it difficult to obtain a clear picture of how AR is perceived and performed in a particular study. This resonates with what Bradbury and colleagues [9] describe as 1 of the quality points of AR, namely “action research process and related methods (should be) clearly articulated and illustrated.” The best practices and lessons learned that were extracted from the included studies were seldom mentioned explicitly. Best practices were often hidden in the description of the project, without much reasoning. Similarly, lessons learned were often described as adaptations made during the project or as plans for the future. Although we observed that some lessons learned turned into best practices over time, we think that researchers could benefit more from each other’s work by providing concrete recommendations. This review is a step in that direction. Both aspects show that the reporting of AR studies in eHealth can be improved to show more clearly what eHealth AR projects can look like and help others in setting up such projects with specific recommendations.

### Limitations

Approximately a third of the included papers (14 out of 44) were published more than 10 years ago. This also means that some of the technologies that are described in the older papers are now relatively old. However, this literature review focuses mainly on the AR methodology and lessons learned about doing action research. Therefore, there was no exclusion criterium regarding the publication date of the papers.

The search yielded several PD-related papers. These papers could have been included, given that some definitions of PD are very similar to AR. However, as our aim was to provide an overview of how AR is done, these were excluded as the researchers of these studies themselves did not identify their studies as being related to AR (ie, not referring to, mentioning, or describing AR). Although this offers a clearer picture of how researchers conduct AR, it also creates a potential limitation in that best practices and lessons learned could be enriched from PD literature.

This overview of AR approaches focuses mostly on the interconnectedness among the approaches, without a comprehensive comparison of the content. Comparing the approaches with regard to the specific aspects of AR that they describe would be a review in and of itself, going beyond the scope of this current review. Therefore, we decided to focus on the definitions that the authors themselves provided even when they also cited AR approaches, as these are most likely to reflect their own vision of AR.

### Conclusions

This review illustrates how AR is conducted in eHealth studies. Studies that fulfilled the inclusion criteria mainly took place in western countries and lasted for 2 to 3 years. Different stakeholders were involved, but the most commonly involved groups were health care professionals and patients. As for the methods used, most studies opted for focus groups and interviews. Even though many studies cited the AR approach proposed by Reason and Bradbury [8], their own definitions of AR were often not explicit in terms of how they implemented AR. Future projects should report their AR definition as well as the best practices and lessons learned more clearly. Other recommendations include paying attention toward developing the skill and confidence of the stakeholders, being aware of the changing role of the researcher, frequently evaluating the project, and disseminating results in an understandable manner.

## Appendix

Multimedia Appendix 1 Full overview of all categories and description of settings for each category.

Multimedia Appendix 2 Full list of categories per setting variable.

## Abbreviations

ADR: action design research
AR: action research
HCI: human computer interaction
PAR: participatory action research
PD: participatory design
